# Lornoxicam with Low-Dose Ketamine versus Pethidine to Control Pain of Acute Renal Colic

**DOI:** 10.1155/2019/3976027

**Published:** 2019-03-13

**Authors:** Ayman A. Metry, Neven G. Fahmy, George M. Nakhla, Rami M. Wahba, Milad Z. Ragaei, Fady A. Abdelmalek

**Affiliations:** Department of Anesthesia, ICU and Pain Management, Faculty of Medicine, Ain Shams University, Cairo, Egypt

## Abstract

**Objectives:**

This study was established to compare single-dose lornoxicam 8 mg (NSAID) in addition to 0.15 mg.kg^−1^ ketamine with single-dose pethidine 50 mg, both administered intravenously (IV), on the quickness and extent of analgesia, disadvantage, and consequence on utilitarian situation.

**Patients and Methods:**

One hundred and twenty patients with acute renal colic pain received in emergency room were included in this prospective, randomized, and double blind clinical study. They were aimlessly designated into one of two groups using a computer-generated table. Group L received lornoxicam 8 mg IV plus 0.15 mg.kg^−1^ ketamine and Group P received pethidine 50 mg IV. Parameters were noticed at baseline and after 0, 15, 30, and 45 minutes and 1 hour after drug administration. The efficiency of the drug was determined by observing: patient rated pain, time to pain relief, rate of pain recurrence, the need for rescue analgesia, adverse events, and functional status.

**Results:**

The visual analogue scale was significantly lower in Group L after 30 minutes in comparison to Group P. In addition, there was statistically significant increase in Group P regarding their need for rescue analgesia after 30 min in comparison to Group L. Group P showed nonsignificantly increased sedation score compared to Group L.

**Conclusion:**

Patients receiving lornoxicam-ketamine attained greater reduction in pain scores and less side effects with better functional state and also are less likely to require further analgesia than those administered pethidine to control acute renal colic pain.

## 1. Introduction

Renal colic is represented by an abrupt attack of severe agonizing pain transmitted from the flank to the groin. Movement of renal calculi over the urinary tract is considered the most prevalent reason for this colic [1].

Impediment of urinary flow takes place with consequent rise in the wall tension provocating prostaglandin (PGs) synthesis in renal pelvis; the latter induces vasodilatation which further raises dieresis and ensuing increase in intrarenal pressure. Also prostaglandins deed precisely on the ureter creating spasm in smooth muscles [2].

Both opioids and nonsteroidal anti-inflammatory drugs deliberated the gold standard for pain relief in acute renal colic [3, 4].

Although opioids are cheap and potent and can be easily titrated but they have various adverse effects as constipation, drowsiness, nausea, and vomiting, larger doses lead to depression of respiration, hypotension, and drug seeking behavior presenting as renal colic [5].

Numerous studies have proved that NSAIDs are competent in managing renal colic [6, 7]. They inhibit synthesis and release of prostaglandins which is the leading cause of pain [8].

Lornoxicam-oxicam derivative is as efficient as opioid in alleviating postoperative pain. It has an encouraging sustainability profile and tolerable gastrointestinal and renal adverse effects [9].

Ketamine is a hydrosoluble aryl-cyclo-alkyl amine which exerts its action by acting mainly on N-Methyl-D-Aspartate receptors (NMDA), non-NMDA receptors, and glutamate binding sites. Ketamine opposes NMDA receptor inducing amnesia, analgesia, psych sensory effects, and neuroprotection [10].

It was reported that low-dose ketamine has tremendous affinity for the NMDA receptor generating suppression of nociception [11, 12].

The present study was conducted to compare the relative advantages and hazards of lornoxicam plus ketamine and pethidine to decide which type of drug is most pertinent for pain control in acute renal colic.

## 2. Patients and Methods

This study was conducted in Ain Shams Specialized Hospital in the period between January 2016 and July 2017 on 120 patients having acute pain of renal origin based on classic clinical history and consistent radiological investigations. The study protocol was approved by institution's ethical committee. This study is registered in ClinicalTrials.com ID NCT03780556.

Patients of either sex aged 20–60 years, who did not administer any analgesics at least within the last two hours, were contained in the study. Exclusion criteria involved patients with liver and renal failure, previous renal surgery, hypersensitivity to lornoxicam, ketamine and pethidine, history of peptic ulcer, gastrointestinal bleeding and perforation, hypertension and history of cardiac diseases, pregnancy and lactation, and urine analysis displaying more than 5 leukocytes suggestive of pyuria.

The proposed study is a prospective, randomized, and double blind. Drug solution was given intravenously to all patients by a nurse who had no idea about the study protocol. Observation of different parameters was done by a doctor who also has no information about the drugs administered.

Patients were randomly assigned into two groups 60 patients each. Group L received lornoxicam (Xefo^R^) 8 mg IV in addition to ketamine (Ketalar^R^) 0.15 mg.kg^−1^ infused in 50 ml normal saline over 10 minutes and Group P received pethidine (pethidine hydrochloride, Fresenius Kabi) 50 mg IV infusion in 50 mL normal saline and infused over 10 minutes.

The parameters used to approach the efficacy of drugs were pain levels using visual analogue score (VAS), onset and duration of action of the drug administered, the need for rescue medications, rate of pain recurrence, physiologic parameters, adverse effects, and functional status.

Respiratory rate, blood pressure, heart rate, oxygen saturation, and sedation score were registered. VAS was used to measure pain intensity. The patients marked their pain severity on a nondemarcated 100 mm horizontal line. This line had “most severe pain” written to the right and “no pain” to the left.

Adverse reactions were listed on datasheet including major and minor adverse effects. Major adverse effects are respiratory depression, renal failure, hypotension, and gastrointestinal bleeding. Minor adverse effects are pain and gastrointestinal disturbance without bleeding as vomiting and diarrhea in addition to dizziness and sleepiness.

Rescue analgesia was given at 30 minutes if on the verbal pain score “no pain” or “pain much better” is not achieved. The rescue drug was pethidine 25 mg slowly IV to be repeated as needed every 15 minutes in an open fashion.

Functional status was calculated as follows, patient's ability to regain regular activities was assessed on 3-point scale: not impaired (capable of coming again to work and driving a car), mildly impaired (able to practice activities of daily living at home), or severely impaired (restricted to bed).

By assuming that pethidine would diminish renal colic pain by 60%, a power calculation with *α* = 0.05 and *β* = 0.80, so, the number of patients required in each group will be at least 58 patients. That was the main reason to enroll 120 patients in this study.

The statistical analysis was executed using a standard SPSS 20 (IBM, Armonk, NY, USA). Normally distributed numerical data are conferred as mean ± SD and differences between groups were compared using the independent Student's* t*-test. Data not normally distributed were compared using Mann-Whitney* U* test and are presented as median (IQR). P < 0.05 is considered statistically significant.

## 3. Results

Prospective, randomized, double blind clinical study containing 120 patients with acute renal pain were received in emergency department. They were randomly assigned to one of two equal groups using a computer-generated table. Group L included 60 patients who received lornoxicam 8 mg IV plus ketamine 0.15 mg.kg^−1^. Group P consisted of 60 patients who received pethidine 50 mg IV. Parameters were checked at baseline and after 15, 30…..and 4 hours of drug treatment. The efficiency of the drug was assessed by observing patient rated pain, time to pain relief, rate of pain recurrence, the need for rescue analgesia, adverse events, and functional status. There was no statistical difference between the 2 groups regarding sex, age, and body weight (Table 1).

As regards heart rate, there was no significant difference between both groups at 0 min and 15 min, while, at 30 min, heart rate was significantly higher in Group P than in Group L (Table 2).

There were no significant differences as regards systolic and diastolic blood pressure in both groups at 0 min, 15 min, and 30 min after drug administration (Table 3) and the same results obtained also as regards respiratory rate (Table 4).

The group of patients received pethidine showed nonsignificant increase in sedation score at 0 min, 15 min, and 30 min after drug administration when compared to the group of patients received lornoxicam-ketamine (Table 5).

There was statistically significant difference between Group P and Group L regarding decrease in oxygen saturation after receiving the drugs, showing a significant decrease in patients who received pethidine compared to those who received lornoxicam (Table 6).

**Figure 1:**
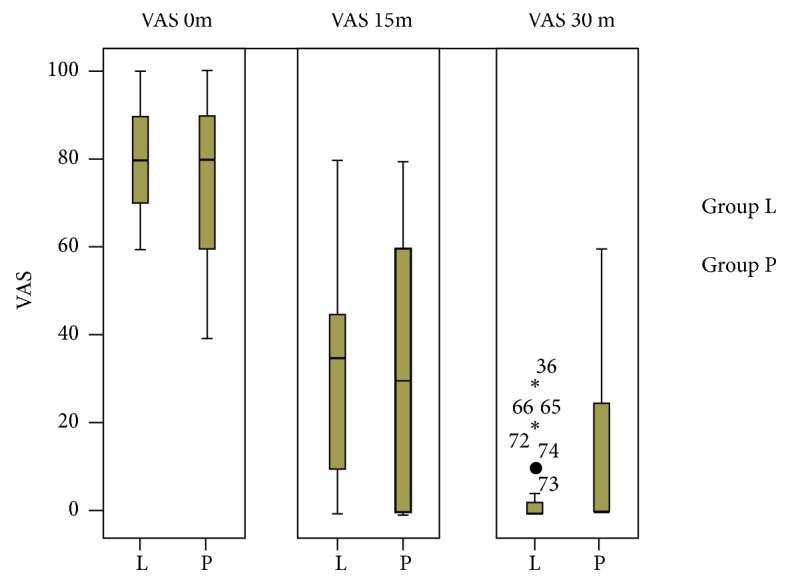
VAS in both groups at 0, 15, and 30 min.

The visual analogue scale was significantly lower in patients who received lornoxicam-ketamine after 30 minutes, while it was higher but without significant difference at 0 min and 15 min after drug administration (Table 7, Figure 1).

There was statistically significant difference between the two groups regarding side effects, which were higher in Group P than in Group L (Table 8).

After 30 minutes, there was statistically significant increase in Group P regarding their need for rescue analgesia, which was higher in Group P than in Group L (Table 9).

There is no statistically significant difference between the two groups regarding their functional state after the 30 minutes (Table 9).

## 4. Discussion

Renal colic is considered one of the most intense pains to be confronted in human life. It is regularly faced in emergency room. It involves 1-5% of the population in modern countries with maximum extent in third to fourth decade of life [13].

Movement of stone through the ureter is the most frequent reason of this pain which radiates from the flanks to the groin and accomplished by nausea, vomiting, and microscopic hematuria [1, 14].

Men are more affected than women with kidney stone disease especially during adulthood with peak in third and fourth decade of life [13].

Renal colic pain elicited as a result of blockage of the urinary flow by a kidney stone and elevated pressure on the urinary tract wall. Smooth muscle spasm arises with edema and inflammation adjacent to the stone and potentiates peristalsis. PGs synthesis and release is aroused by tension in renal pelvis which in turn induce dieresis and vasodilatation. The explicit outcome of PGs on the ureter leads to spasm in the smooth muscles of the ureteric wall [15]. The major goal of emergency department is to alleviate pain until either spontaneous passage or surgical interference.

The rate of using NSAIDs is uprising rather than opioids in management of acute renal colic; recent studies have concluded that these drugs were to be as potent as opioids [16, 17].

In the literature there are huge numbers of controlled studies comparing the competency and safety of NSAIDs and opioids. Many clinical trials have found that NSAIDs and opioids produce equal standards of postoperative analgesia but opioids generated higher rates of dizziness, nausea, and vomiting [18, 19].

Identical results have been proved in those with acute biliary colic and limb injuries [20, 21].

Lornoxicam (chlortenoxicam) is a NSAID with strong analgesic and anti-inflammatory effect in addition to equal cyclooxygenase (COX-1/COX-2) inhibition and better gastrointestinal and tolerability profile. This is as a result of its short half-life (~4 hs) in comparison to more than 24 h for the other NSAIDs [22, 23]. Lornoxicam varies from other oxicam compounds in its vigorous prohibition of prostaglandin biosynthesis, a characteristic that justifies the specifically marked potency of the drug. Lornoxicam differs from other NSAIDs in that its suppression of cyclooxygenase does not produce rise in leukotriene production, signifying that arachidonic acid is not changed to the 5-lipoxygenase cascade, owing to reduction in occurrence of adverse events [24].

Ilias et al. reported that lornoxicam 8 mg IV was higher than placebo and equal to tramadol 50 mg IV in controlling moderate to severe posthysterectomy pain [25]. Işik et al. concluded that 8 mg lornoxicam administered preoperatively was more effective than tramadol 50 mg in controlling early postoperative tonsillectomy pain in adult patients [26]. Also Staunstrup et al. compared the analgesic efficacy of a single dose of intramuscular lornoxicam 16 mg and tramadol 100 mg in 76 patients after anterior cruciate ligament arthroscopic reconstruction and stated that lornoxicam is an efficient alternative to tramadol for alleviating moderate to severe pain [27].

Lornoxicam 16 mg was found to be equal to fentanyl as intraoperative IV analgesia and was combined with fewer incidences of adverse events [28]; however, it is more potent in inhibiting early postoperative pain in patients undergoing minor to moderate day-case surgical procedures.

Low-dose ketamine has great tendency for the NMDA receptor resulting in suppression of nociception [11, 12]. It is postulated that low-dose ketamine may inhibit more specifically NMDA receptors, while full-anesthetic dose of ketamine potentiates distinct types of opioid receptors with various tendencies (*μ*, *κ*, and *σ* opioid receptors) [29, 30].

Many studies showed that combination of morphine with low-dose ketamine in patients with moderate to severe acute pain diminish morphine demands by about 26%–60% [31–35]. Johansson et al. conclude that only half as much rescue morphine was given to patients administering 0.2 mg.kg^−1^ ketamine when compared to control group which used morphine [36].

Ketamine administration at doses of only 0.15–0.3 mg.kg^−1^ was found in many studies to cause extensive neuropsychiatric adverse reactions. To avoid such undesirable event, a very low-concentration intermittent intravenous bolus or a 2–3 mcg.kg^−1^.min^−1^ continuous infusion might be appreciated [33, 34]. Also, Motov et al. concluded that low-dose ketamine given as a short infusion is accomplished with significantly lower rates of sedation and sensation of unreality with no difference in analgesic efficacy when compared to intravenous bolus administration. Use of low-dose ketamine (0.1–0.3 mg.kg^−1^ IV) has been exhibited to be opioid sparing. Some of the main effects with IV push low-dose ketamine consist of its adverse reactions such as dizziness, nausea, vomiting, and feelings of unreality [37].

We preferred to administer ketamine by infusion over 10 minutes to avoid unwanted side effects like nausea, vomiting, and feeling of unreality. Mild sedation occurring with this low-dose ketamine is wanted effect in patients with acute renal colic.

Ketamine is metabolized mainly to norketamine (80%) which is an effective metabolite. Norketamine commences to appear in blood 2–3 min after a ketamine IV bolus injection and approaches a peak about 30 min later. Norketamine is known to have an analgesic effect, whose potency is about 20–30% in comparison to ketamine [37]. Norketamine is slowly eliminated from circulation and continues more than 5 h after administration [38]. Norketamine lasts in circulation for longer time than ketamine because ketamine has less elimination half time than norketamine [39]. As a result of norketamine accumulation, the demand for ketamine, when given in continuous perfusion, diminishes over time [40].

Pethidine is a synthetic opioid pain medication of the phenylpiperidine class, synthesized in 1939 as a potential anticholinergic agent [40–42]. It applies its analgesic effects, like morphine, by working as an agonist at the *μ*-opioid receptor [42]. It has a more rapid onset of action than morphine which is related to its higher lipid-solubility.

It was thought that pethidine has many advantages over morphine especially in controlling pain due to biliary spasm and renal colic as a consequence to its anticholinergic effect, but later researches denied this and considered it as more toxic than other opioids due to its metabolite norpethidine chiefly with long term application. Pethidine could lead to serotonin syndrome due to serotonergic effects of its norpethidine metabolite [43, 44]. Nowadays many literatures and centers try to hinder pethidine use and replace it with more safe pain killer.

We performed our study to compare the analgesic effect of pethidine 50 mg, as it is still the most common pain killer used in our country as a single dose of 50 mg administered to all adult patients to control renal colic in emergency room, with lornoxicam which is a short acting NSAID stated to be efficient in controlling acute pain.

It was more reasonable if we administered pethidine according to the body weight or with titration according to the patient's pain feeling but we concentrated to mimic what happens really in emergency room for management of acute renal colic pain.

Low-dose ketamine added to lornoxicam to potentiate the analgesic effect and at the same time may help in mild sedation which is obtained from pethidine and deficient with lornoxicam and desired in such patients.

Many researches nowadays try to contribute low-dose ketamine to control acute pain control in emergency room to get benefit from its analgesic effect and at the same time to spare narcotics as much as possible [45–47].

In our study we decided also to add pethidine to 50 ml normal saline and infuse over 10 minutes to reduce side effects from direct bolus injection like nausea, vomiting, and histamine release especially at site of injection, in addition to, similarity in the way of administration to ketamine.

We failed to find studies which compare IV lornoxicam to IV pethidine. We showed that IV lornoxicam in addition to low-dose ketamine contributed to slightly better analgesia with fewer disadvantages than IV pethidine in spite of the finding of enhanced analgesia in patients with renal colic may be related to local synthesis and production of prostaglandins particular to this situation.

In a previous study [48] Cordell and colleagues stated that single-dose IV ketorolac 60 mg had superior early analgesia than single-dose IV pethidine 50 mg which supports our results.

### 4.1. Limitations

Our study had some limitations; as there were no placebo control group and this was for ethical reasons, so comparison was done using an active control only. Adverse effects of drugs were measured for short time because the study duration was 4 hours only. As many emergency department (ED) studies, we used a convenience sample and this may have introduced a selection bias. Finally, the included trials used fixed dose of pethidine rather than titration to an appropriate level of pain relief. The standard practice in the majority of emergency department is titration rather than fixed dose and this limits the applicability of our results to everyday practice [48].

## 5. Conclusion

In the doses studied, single-dose lornoxicam (8mg IV) in addition to ketamine 0.15 mg.kg^−1^ is as effective as pethidine (50 mg IV) for the ED relief of acute renal colic. In addition, IV lornoxicam causes greater reduction in pain scores, less side effects with better functional state, and also are less likely to require further analgesia than IV pethidine.

## Figures and Tables

**Table 1:** Demographic data.

Group	Group I	Group II	P value
Number	60	60	
Sex (F/M)	(22/38)	(20/40)	0.701
Age (years)	37.8±12.8	39.8±11.3	0.366
Body weight (Kg)	73.34±13.2	72.75±14.6	0.816

P > 0.05 is considered statistically nonsignificant.

**Table 2:** Heart rate in both groups.

	Group L (n=60)	Group P (n=60)	p-value
0 min	84.6 ± 9.2	85.37 ± 12.8	0.67
15 min	84.08 ± 9.87	84.1 ± 15.39	0.994
30 min	79.13 ± 7.94	85.7 ± 13.31^*∗*^	0.0013

Data are presented as mean ±SD.

^*∗*^P< 0.05 is considered statistically significant.

**Table 3:** Systolic and diastolic blood pressure in both groups.

	Group L (n=60)	Group P (n=60)	p-value
*0m*			
Systolic	118.33 ± 16.2	117.8 ± 20.1	0.88
Diastolic	75.33 ± 11.27	76.5 ± 14.36	0.62

*15m*			
Systolic	112.4 ± 14.3	115.7 ± 17.4	0.254
Diastolic	71.5 ± 9.12	74.83 ± 11.57	0.068

*30m*			
Systolic	111.17 ± 13.67	117.8 ± 16.1	0.202
Diastolic	71 ± 8.77	73.5 ± 11.2	0.175

Data are presented as mean ±SD.

P> 0.05 is considered statistically nonsignificant.

So, there is no significant difference between the 2 groups.

**Table 4:** Respiratory rate in both groups.

	Group L (n=60)	Group P (n=60)	p-value
0m	21. 48 ±3.68	20.24± 4.65	0. 097
15m	20.28± 2.57	19.7± 3.28	0. 31
30m	19.07± 2.44	19.7± 3.69	0.245

Data are presented as mean ±SD.

P> 0.05 is considered statistically nonsignificant.

So, no statistically significant difference between the two groups.

**Table 5:** Sedation score in both groups.

	Group L (n=60)	Group P (n=60)	p-value
0m	0	0	0
15m	2.64 ± 1.29	2.83 ± 1.36	0.215
30m	2.4 ± 0. 65	2.43± 0.72	0.253

Data are presented as mean ±SD.

P> 0.05 is considered statistically nonsignificant.

**Table 6:** Oxygen saturation in both groups.

	Group L (n=60)	Group P (n=60)	p-value
0m	99.55± 0.746	99.64± 0.3	0.496
15m	99.87± 0.343	98.65± 0.1^*∗*^	0.004
30m	99.8± 0.1	99.07± 0.4^*∗*^	0.004

Data are presented as mean ±SD.

P< 0.05 is considered statistically significant.^*∗*^

**Table 7:** Visual analogue scale in both groups.

	Group L (n=60)	Group P (n=60)	p-value
0m	80(70-90)	80(60-90)	0.198
15m	35(5-47)	30(0-60)	0.879
30m	0(0-2.75)	0( 0-40)^*∗*^	0.021

Data are presented as median (IQR).

^*∗*^P < 0.05 is considered statistically significant.

**Table 8:** Side effects in both groups.

	Group L (n=60)	Group P (n=60)	p-value
0m	0	0	0

15m			< 0.001
1	3	0	
2	0	6	
3	0	7	

30m			0.003
1	6	0	
2	0	6	
3	0	7	

Data are presented as number of patients.

^*∗*^P< 0.05 is considered statistically significant.

**Table 9:** Rescue analgesia and functional scale in both groups.

	Group L (n = 60)	Group P (n = 60)	p-value
Rescue analgesia	6	16^*∗*^	0. 032
Function Scale	1(1-1)	1(1-2)	0.196

Data are presented as median (IQR) or number of patients.

^*∗*^P< 0.05 is considered statistically significant.

## Data Availability

The data used to support the findings of this study are available from the corresponding author upon request.
